# 3-Chloro­phenyl 4-methyl­benzoate

**DOI:** 10.1107/S1600536808019351

**Published:** 2008-07-05

**Authors:** B. Thimme Gowda, Sabine Foro, K. S. Babitha, Hartmut Fuess

**Affiliations:** a Department of Chemistry, Mangalore University, Mangalagangotri 574 199, Mangalore, India; b Institute of Materials Science, Darmstadt University of Technology, Petersenstrasse 23, D-64287 Darmstadt, Germany

## Abstract

The crystal structure of the title compound 3CP4MBA, C_14_H_11_ClO_2_, resembles those of 3-methyl­phenyl 4-methyl­benzoate (3MP4MBA), 4-methyl­phenyl 4-methyl­benzoate (4MP4MBA), 4-methyl­phenyl 4-chloro­benzoate (4CP4MBA) and other aryl benzoates with similar bond parameters. The dihedral angle between the benzene rings in 3CP4MBA is 71.75 (7)°, compared with 56.82 (7)° in 3MP4MBA and 63.57 (5)° in 4MP4MBA. In the crystal structure, the mol­ecules are aligned with their long axis approximately along the [101] direction and stacked along the *c* axis.

## Related literature

For related literature, see: Gowda *et al.* (2007 ▶, 2008 ▶); Nayak & Gowda (2008 ▶).
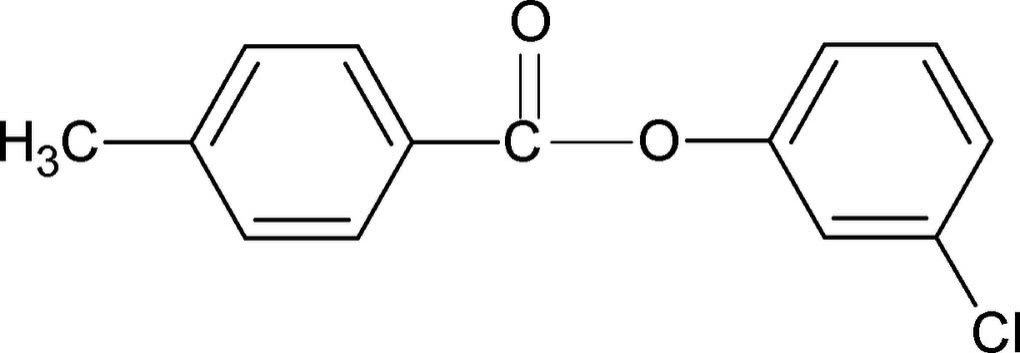


## Experimental

### 

#### Crystal data

C_14_H_11_ClO_2_
*M* *_r_* = 246.68
Monoclinic, 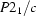

*a* = 13.706 (2) Å
*b* = 12.142 (2) Å
*c* = 7.3807 (5) Å
β = 100.625 (9)°
*V* = 1207.2 (3) Å^3^
*Z* = 4
Cu *K*α radiation
μ = 2.69 mm^−1^
*T* = 299 (2) K
0.50 × 0.27 × 0.10 mm

#### Data collection

Enraf–Nonius CAD-4 diffractometer
Absorption correction: ψ scan (North *et al.*, 1968 ▶) *T*_min_ = 0.344, *T*_max_ = 0.767
4283 measured reflections
2146 independent reflections
1801 reflections with *I* > 2σ(*I*)
*R*_int_ = 0.033
3 standard reflections frequency: 120 min intensity decay: 1.0%

#### Refinement

*R*[*F* ^2^ > 2σ(*F* ^2^)] = 0.038
*wR*(*F* ^2^) = 0.111
*S* = 1.04
2146 reflections
179 parameters
H atoms treated by a mixture of independent and constrained refinement
Δρ_max_ = 0.19 e Å^−3^
Δρ_min_ = −0.28 e Å^−3^

### 

Data collection: *CAD-4-PC* (Enraf–Nonius, 1996 ▶); cell refinement: *CAD-4-PC*; data reduction: *REDU4* (Stoe & Cie, 1987 ▶); program(s) used to solve structure: *SHELXS97* (Sheldrick, 2008 ▶); program(s) used to refine structure: *SHELXL97* (Sheldrick, 2008 ▶); molecular graphics: *PLATON* (Spek, 2003 ▶); software used to prepare material for publication: *SHELXL97*.

## Supplementary Material

Crystal structure: contains datablocks I, global. DOI: 10.1107/S1600536808019351/hk2479sup1.cif

Structure factors: contains datablocks I. DOI: 10.1107/S1600536808019351/hk2479Isup2.hkl

Additional supplementary materials:  crystallographic information; 3D view; checkCIF report

## Figures and Tables

**Fig. 1.**
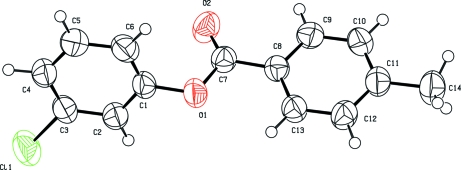
Molecular structure of the title compound, showing the atom labeling. Displacement ellipsoids are drawn at the 50% probability level. H atoms are represented as small spheres of arbitrary radius.

**Fig. 2.**
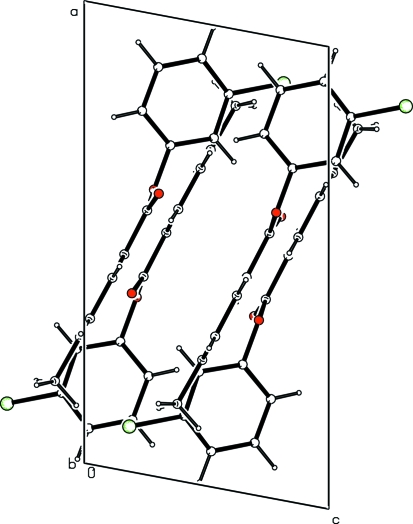
Molecular packing of the title compound.

## supplementary crystallographic information

### Comment

In the present work, as part of a study of the substituent effects on the solid state geometries of aryl benzoates (Gowda *et al.*, 2007, 2008), the structure of 3-chlorophenyl 4-methylbenzoate (3CP4MBA) has been determined. The structure of 3CP4MBA (Fig. 1) is similar to those of 3-methylphenyl 4-methyl- benzoate (3MP4MBA), 4-methylphenyl 4-methylbenzoate (4MP4MBA), 4-methylphenyl 4-chlorobenzoate (4MP4CBA) and other aryl benzoates (Gowda *et al.*, 2007, 2008). The bond parameters in 3CP4MBA are similar to those in 3MP4MBA, 4MP4MBA, 4CP4MBA and other aryl benzoates. The dihedral angle between the benzene and phenyl rings in 3CP4MBA is 71.75 (7)°, compared to the values of 56.82 (7)° in 3MP4MBA and 63.57 (5)° in 4MP4MBA. In the crystal structure, the molecules are elongated approximatelly along the [101] direction and stacked along the c axis (Fig. 2).

### Experimental

The title compound was prepared according to a literature method (Nayak & Gowda, 2008). The purity of the compound was checked by determining its melting point. It was characterized by recording its infrared and NMR spectra (Nayak & Gowda, 2008). Single crystals of the title compound were obtained by slow evaporation of its ethanolic solution.

### Refinement

H atoms (for CH) were located in difference map and refined [ C-H = 0.89 (2) -0.98 (2) Å; U_iso_(H) = 0.067-0.079 Å^2^]. The methyl H atoms were positioned geometrically, with C-H= 0.96 Å, and constrained to ride on the parent atom, with U_iso_(H) = 1.2U_eq_(C).

### Crystal data

**Table:**

C_14_H_11_ClO_2_	*F*_000_ = 512
*M**_r_* = 246.68	*D*_x_ = 1.357 Mg m^−^^3^
Monoclinic, *P*2_1_/*c*	Cu *K*α radiation λ = 1.54180 Å
Hall symbol: -P 2ybc	Cell parameters from 25 reflections
*a* = 13.706 (2) Å	θ = 4.9–22.0º
*b* = 12.142 (2) Å	µ = 2.69 mm^−^^1^
*c* = 7.3807 (5) Å	*T* = 299 (2) K
β = 100.625 (9)º	Plate, colorless
*V* = 1207.2 (3) Å^3^	0.50 × 0.27 × 0.10 mm
*Z* = 4	

### Data collection

**Table:**

Enraf–Nonius CAD-4 diffractometer	*R*_int_ = 0.033
Radiation source: fine-focus sealed tube	θ_max_ = 67.0º
Monochromator: graphite	θ_min_ = 3.3º
*T* = 299(2) K	*h* = −16→16
ω/2θ scans	*k* = −14→0
Absorption correction: ψ scan(North *et al.*, 1968)	*l* = −8→8
*T*_min_ = 0.344, *T*_max_ = 0.767	3 standard reflections
4283 measured reflections	every 120 min
2146 independent reflections	intensity decay: 1.0%
1801 reflections with *I* > 2σ(*I*)	

### Refinement

**Table:**

Refinement on *F*^2^	Hydrogen site location: inferred from neighbouring sites
Least-squares matrix: full	H atoms treated by a mixture of independent and constrained refinement
*R*[*F*^2^ > 2σ(*F*^2^)] = 0.038	*w* = 1/[σ^2^(*F*_o_^2^) + (0.0556*P*)^2^ + 0.2388*P*] where *P* = (*F*_o_^2^ + 2*F*_c_^2^)/3
*wR*(*F*^2^) = 0.111	(Δ/σ)_max_ = 0.004
*S* = 1.04	Δρ_max_ = 0.19 e Å^−^^3^
2146 reflections	Δρ_min_ = −0.28 e Å^−^^3^
179 parameters	Extinction correction: SHELXL97 (Sheldrick, 2008), Fc^*^=kFc[1+0.001xFc^2^λ^3^/sin(2θ)]^-1/4^
Primary atom site location: structure-invariant direct methods	Extinction coefficient: 0.0200 (13)
Secondary atom site location: difference Fourier map	

### Special details

**Table:**

Geometry. All e.s.d.'s (except the e.s.d. in the dihedral angle between two l.s. planes) are estimated using the full covariance matrix. The cell e.s.d.'s are taken into account individually in the estimation of e.s.d.'s in distances, angles and torsion angles; correlations between e.s.d.'s in cell parameters are only used when they are defined by crystal symmetry. An approximate (isotropic) treatment of cell e.s.d.'s is used for estimating e.s.d.'s involving l.s. planes.
Refinement. Refinement of F^2^ against ALL reflections. The weighted R-factor wR and goodness of fit S are based on F^2^, conventional R-factors R are based on F, with F set to zero for negative F^2^. The threshold expression of F^2^ > 2sigma(F^2^) is used only for calculating R-factors(gt) etc. and is not relevant to the choice of reflections for refinement. R-factors based on F^2^ are statistically about twice as large as those based on F, and R- factors based on ALL data will be even larger.

### Fractional atomic coordinates and isotropic or equivalent isotropic displacement parameters (Å^2^)

**Table:**

	*x*	*y*	*z*	*U*_iso_*/*U*_eq_	
Cl1	0.09746 (4)	0.45783 (6)	0.18459 (7)	0.0902 (3)	
O1	0.38017 (9)	0.38035 (10)	0.71453 (19)	0.0659 (4)	
O2	0.38682 (9)	0.19643 (10)	0.6950 (2)	0.0690 (4)	
C1	0.27767 (12)	0.37826 (14)	0.6477 (3)	0.0550 (4)	
C2	0.24434 (13)	0.41200 (15)	0.4698 (3)	0.0557 (4)	
H2	0.2874 (15)	0.4294 (17)	0.388 (3)	0.067*	
C3	0.14290 (13)	0.41514 (15)	0.4089 (2)	0.0566 (4)	
C4	0.07707 (14)	0.38530 (16)	0.5193 (3)	0.0612 (5)	
H4	0.0062 (16)	0.3899 (17)	0.472 (3)	0.073*	
C5	0.11328 (15)	0.35196 (18)	0.6970 (3)	0.0655 (5)	
H5	0.0675 (17)	0.3304 (19)	0.779 (3)	0.079*	
C6	0.21440 (15)	0.34873 (16)	0.7635 (3)	0.0627 (5)	
H6	0.2394 (16)	0.3273 (18)	0.878 (3)	0.075*	
C7	0.42774 (12)	0.28135 (14)	0.7426 (2)	0.0518 (4)	
C8	0.53187 (12)	0.29444 (13)	0.8356 (2)	0.0492 (4)	
C9	0.58762 (14)	0.20077 (15)	0.8849 (3)	0.0581 (5)	
H9	0.5605 (15)	0.1327 (19)	0.856 (3)	0.070*	
C10	0.68465 (14)	0.20926 (17)	0.9760 (3)	0.0631 (5)	
H10	0.7217 (16)	0.1486 (19)	1.014 (3)	0.076*	
C11	0.72884 (13)	0.31058 (17)	1.0174 (3)	0.0602 (5)	
C12	0.67287 (14)	0.40421 (17)	0.9643 (3)	0.0614 (5)	
H12	0.7006 (16)	0.4749 (19)	0.989 (3)	0.074*	
C13	0.57564 (13)	0.39698 (15)	0.8758 (3)	0.0561 (4)	
H13	0.5378 (15)	0.4611 (17)	0.839 (3)	0.067*	
C14	0.83424 (15)	0.3191 (2)	1.1193 (3)	0.0819 (7)	
H14A	0.8782	0.3245	1.0322	0.098*	
H14B	0.8506	0.2549	1.1944	0.098*	
H14C	0.8412	0.3835	1.1961	0.098*	

### Atomic displacement parameters (Å^2^)

**Table:**

	*U*^11^	*U*^22^	*U*^33^	*U*^12^	*U*^13^	*U*^23^
Cl1	0.0729 (4)	0.1236 (6)	0.0670 (4)	0.0106 (3)	−0.0054 (2)	0.0248 (3)
O1	0.0505 (7)	0.0512 (7)	0.0862 (9)	−0.0002 (5)	−0.0126 (6)	0.0011 (6)
O2	0.0537 (7)	0.0552 (8)	0.0950 (10)	−0.0058 (6)	0.0059 (7)	−0.0148 (7)
C1	0.0483 (9)	0.0433 (9)	0.0673 (10)	0.0012 (7)	−0.0055 (8)	−0.0030 (8)
C2	0.0517 (9)	0.0516 (9)	0.0619 (10)	0.0013 (8)	0.0050 (8)	0.0000 (8)
C3	0.0541 (9)	0.0547 (10)	0.0566 (10)	0.0075 (8)	−0.0011 (8)	0.0029 (8)
C4	0.0496 (9)	0.0574 (11)	0.0735 (12)	0.0050 (8)	0.0031 (9)	0.0024 (9)
C5	0.0618 (11)	0.0634 (11)	0.0720 (12)	0.0015 (9)	0.0139 (9)	0.0075 (10)
C6	0.0679 (11)	0.0571 (11)	0.0583 (10)	0.0017 (9)	−0.0006 (9)	0.0054 (9)
C7	0.0502 (9)	0.0519 (9)	0.0525 (9)	−0.0013 (8)	0.0071 (7)	−0.0023 (7)
C8	0.0482 (9)	0.0513 (9)	0.0477 (8)	0.0003 (7)	0.0074 (7)	−0.0004 (7)
C9	0.0559 (10)	0.0495 (10)	0.0688 (11)	−0.0001 (8)	0.0111 (8)	0.0004 (9)
C10	0.0533 (10)	0.0620 (11)	0.0733 (12)	0.0108 (9)	0.0096 (9)	0.0114 (9)
C11	0.0487 (9)	0.0764 (12)	0.0543 (10)	0.0012 (8)	0.0062 (7)	0.0041 (8)
C12	0.0553 (10)	0.0593 (11)	0.0659 (11)	−0.0069 (9)	0.0016 (8)	−0.0070 (9)
C13	0.0530 (9)	0.0496 (10)	0.0622 (10)	0.0020 (8)	0.0016 (8)	−0.0026 (8)
C14	0.0545 (11)	0.1051 (18)	0.0804 (14)	−0.0022 (11)	−0.0028 (10)	0.0104 (13)

### Geometric parameters (Å, °)

**Table:**

C1—C2	1.371 (3)	C8—C9	1.381 (2)
C1—C6	1.373 (3)	C8—C13	1.390 (2)
C1—O1	1.401 (2)	C9—C10	1.379 (3)
C2—C3	1.381 (2)	C9—H9	0.91 (2)
C2—H2	0.94 (2)	C10—C11	1.380 (3)
C3—C4	1.371 (3)	C10—H10	0.91 (2)
C3—Cl1	1.7372 (18)	C11—C12	1.387 (3)
C4—C5	1.375 (3)	C11—C14	1.504 (3)
C4—H4	0.97 (2)	C12—C13	1.374 (3)
C5—C6	1.383 (3)	C12—H12	0.94 (2)
C5—H5	0.98 (2)	C13—H13	0.95 (2)
C6—H6	0.89 (2)	C14—H14A	0.9600
C7—O2	1.195 (2)	C14—H14B	0.9600
C7—O1	1.365 (2)	C14—H14C	0.9600
C7—C8	1.474 (2)		
			
C2—C1—C6	122.49 (17)	C13—C8—C7	122.60 (15)
C2—C1—O1	117.87 (17)	C10—C9—C8	120.27 (17)
C6—C1—O1	119.54 (17)	C10—C9—H9	119.7 (13)
C1—C2—C3	117.26 (18)	C8—C9—H9	120.1 (13)
C1—C2—H2	122.8 (13)	C9—C10—C11	121.26 (18)
C3—C2—H2	119.9 (13)	C9—C10—H10	121.6 (14)
C4—C3—C2	122.20 (17)	C11—C10—H10	117.1 (14)
C4—C3—Cl1	119.04 (14)	C10—C11—C12	118.08 (17)
C2—C3—Cl1	118.76 (15)	C10—C11—C14	120.93 (18)
C3—C4—C5	118.89 (17)	C12—C11—C14	120.98 (19)
C3—C4—H4	119.9 (12)	C13—C12—C11	121.28 (18)
C5—C4—H4	121.2 (13)	C13—C12—H12	118.1 (13)
C4—C5—C6	120.6 (2)	C11—C12—H12	120.6 (14)
C4—C5—H5	120.3 (13)	C12—C13—C8	120.07 (17)
C6—C5—H5	119.1 (13)	C12—C13—H13	121.0 (13)
C1—C6—C5	118.57 (18)	C8—C13—H13	118.9 (13)
C1—C6—H6	119.4 (14)	C11—C14—H14A	109.5
C5—C6—H6	122.0 (14)	C11—C14—H14B	109.5
O2—C7—O1	122.00 (15)	H14A—C14—H14B	109.5
O2—C7—C8	126.27 (16)	C11—C14—H14C	109.5
O1—C7—C8	111.73 (14)	H14A—C14—H14C	109.5
C9—C8—C13	119.03 (16)	H14B—C14—H14C	109.5
C9—C8—C7	118.37 (15)	C7—O1—C1	117.23 (13)
			
C6—C1—C2—C3	−0.2 (3)	C13—C8—C9—C10	1.3 (3)
O1—C1—C2—C3	−176.61 (15)	C7—C8—C9—C10	−178.50 (17)
C1—C2—C3—C4	−0.5 (3)	C8—C9—C10—C11	−1.0 (3)
C1—C2—C3—Cl1	179.81 (14)	C9—C10—C11—C12	−0.2 (3)
C2—C3—C4—C5	0.5 (3)	C9—C10—C11—C14	179.1 (2)
Cl1—C3—C4—C5	−179.75 (15)	C10—C11—C12—C13	1.1 (3)
C3—C4—C5—C6	0.1 (3)	C14—C11—C12—C13	−178.1 (2)
C2—C1—C6—C5	0.8 (3)	C11—C12—C13—C8	−0.8 (3)
O1—C1—C6—C5	177.16 (17)	C9—C8—C13—C12	−0.4 (3)
C4—C5—C6—C1	−0.8 (3)	C7—C8—C13—C12	179.41 (17)
O2—C7—C8—C9	−4.5 (3)	O2—C7—O1—C1	7.6 (3)
O1—C7—C8—C9	175.56 (16)	C8—C7—O1—C1	−172.44 (15)
O2—C7—C8—C13	175.72 (19)	C2—C1—O1—C7	−109.35 (18)
O1—C7—C8—C13	−4.2 (2)	C6—C1—O1—C7	74.1 (2)

## References

[bb1] Enraf–Nonius (1996). *CAD-4-PC.* Enraf–Nonius, Delft, The Netherlands.

[bb2] Gowda, B. T., Foro, S., Babitha, K. S. & Fuess, H. (2007). *Acta Cryst.* E**63**, o3867.

[bb3] Gowda, B. T., Svoboda, I., Babitha, K. S. & Fuess, H. (2008). *Acta Cryst.* E**64**, o88. 10.1107/S1600536807062137 PMC2915044 21200965

[bb4] Nayak, R. & Gowda, B. T. (2008). *Z. Naturforsch. Teil A*, **63** In the press.

[bb5] North, A. C. T., Phillips, D. C. & Mathews, F. S. (1968). *Acta Cryst.* A**24**, 351–359.

[bb6] Sheldrick, G. M. (2008). *Acta Cryst.* A**64**, 112–122. 10.1107/S0108767307043930 18156677

[bb7] Spek, A. L. (2003). *J. Appl. Cryst.* **36**, 7–13.

[bb8] Stoe & Cie (1987). *REDU4* Stoe & Cie GmbH, Darmstadt, Germany.

